# JACOP: A simple and robust method for the automated classification of protein sequences with modular architecture

**DOI:** 10.1186/1471-2105-6-216

**Published:** 2005-08-31

**Authors:** Peter Sperisen, Marco Pagni

**Affiliations:** 1 Swiss Institute of Bioinformatics, Computational Cancer Genomics Group – ISREC, Ch. des Boveresses 155, 1066 Epalinges, Switzerland; 2 Swiss Institute of Bioinformatics, Vital IT Group, BEP-UNIL, 1015 Lausanne, Switzerland

## Abstract

**Background:**

Whole-genome sequencing projects are rapidly producing an enormous number of new sequences. Consequently almost every family of proteins now contains hundreds of members. It has thus become necessary to develop tools, which classify protein sequences automatically and also quickly and reliably. The difficulty of this task is intimately linked to the mechanism by which protein sequences diverge, i.e. by simultaneous residue substitutions, insertions and/or deletions and whole domain reorganisations (duplications/swapping/fusion).

**Results:**

Here we present a novel approach, which is based on random sampling of sub-sequences (probes) out of a set of input sequences. The probes are compared to the input sequences, after a normalisation step; the results are used to partition the input sequences into homogeneous groups of proteins. In addition, this method provides information on diagnostic parts of the proteins. The performance of this method is challenged by two data sets. The first one contains the sequences of prokaryotic lyases that could be arranged as a multiple sequence alignment. The second one contains all proteins from Swiss-Prot Release 36 with at least one Src homology 2 (SH2) domain – a classical example for proteins with modular architecture.

**Conclusion:**

The outcome of our method is robust, highly reproducible as shown using bootstrap and resampling validation procedures. The results are essentially coherent with the biology. This method depends solely on well-established publicly available software and algorithms.

## Background

Whole-genome sequencing projects are currently producing an enormous amount of new sequences. As a consequence, protein sequence databases are rapidly increasing in size, thus resulting in severe practical consequences. For example, a simple database search can now yield hundreds of matches. An automated but sensible grouping of those proteins appears as an indispensable solution to analyse such an output in a timely manner.

Proteins are often described as the assembly of several structural/functional units called domains. Isolation of the domain sub-sequences renders a multiple alignment possible, from which domain descriptors are built based on efficient methods for remote homology detection (PSSM [1], generalised profiles [2], hidden Markov models (HMM) [3]). This led to the thriving development of protein-domain databases such as PROSITE [4], Pfam [5], Blocks [6], PRINTS [7], IDENTIFY [8], ProDom [9], Domo [10], SMART [11] and ADDA [12]. Classification of domain sub-sequences is relatively straightforward through direct sequence comparison but does not address the problem of whole multi-domain protein classification.

As a possible alternative to direct sequence comparison, proteins could be classified according to their domain architecture [13,14]. Although no general methodology has yet emerged, many review articles on particular families are available [15-17]. The highly modular proteins that are involved in signalling pathways are a typical example where domain architecture usually is diagnostic for the protein function (such an example is treated below). Unfortunately, in the course of evolution, the linear and modular organisation of proteins is not always preserved because of rare genetic events that are responsible for domain swapping, duplication or deletion. One must realise that the number of these particular cases increases with the number of genome sequences [18-20] and renders automated classification difficult.

Finally, the emerging picture of the human proteome provides evidence that alternative splicing is not anecdotal [21]. Indeed, this mechanism is a potential source of sequence variation and the proper handling of splice variants by clustering protocols is a challenge.

Several recent contributions addressed the clustering of very large sets of possibly unrelated protein sequences (e.g. TRIBE-MCL [22], Picasso [23], Systers [24], COG [25], ProtoMap [26], ProtoNet [27], ClusTr [28] and ProClass [29]). All these approaches are based on pairwise or multiple alignments of the sequences to be analysed. Alternatively alignment-free sequence comparison methods were proposed ([30] and references therein). However, they have not yet been widely used for the clustering of very large sets.

Here we present a novel method called JACOP (just another classification of proteins), which stands somewhere between pairwise alignment methods and alignment-free methods. We will employ a collection of unordered short sub-sequences as an intermediary layer in the comparison of two sequences. As a consequence, the linearity of the domain architecture – present in the investigated protein set – no longer plays an important role. In comparison to existing methods, our protocol is remarkably simple but nevertheless appears to be robust and highly reproducible on difficult test sets.

## Results and discussion

### Protocol

**Figure 1:**
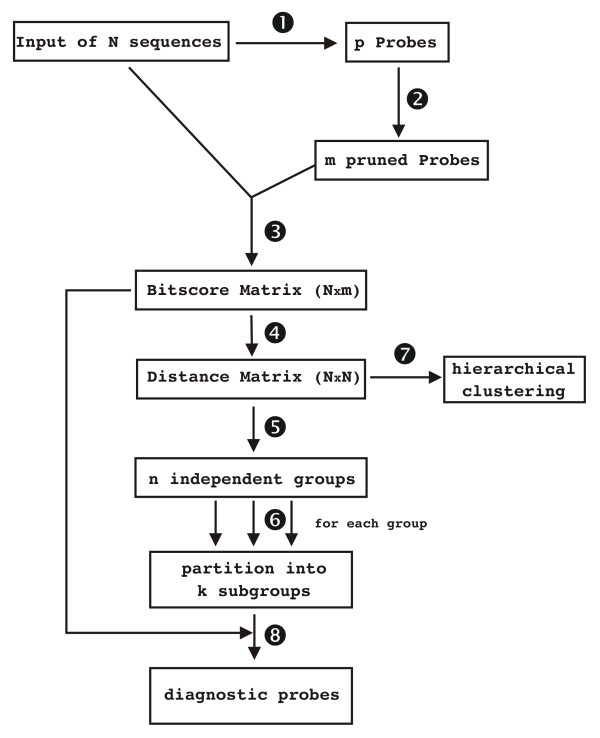
Schematic representation of the reference protocol. The numbers correspond to the steps explained in the main text.

The different steps to partition a set of unaligned protein sequences using the JACOP protocol are presented below and summarised in Figure 1. The parameters used for the reference protocol are those established after intense testing leading to the most consistent results. The rationale behind the selection of these parameters is presented further down. The only prerequisite for a given sequence is a length of at least 50 residues. Otherwise no a priori knowledge about the protein sequences is needed.

#### 1. Random probe generation

p subsequences of 50 residues length (probes) are randomly sampled from the set of N input sequences. All probes that can possibly be generated out of the input sequences are equally likely. The sampling process is carried out until the cumulated length of the probes exceeds three times the cumulated length of the input sequences.

#### 2. Pruning probes

An all-*versus*-all comparison of the probes is performed using the SW algorithm with a Blosum62 [31] similarity matrix and gap opening/extension penalties set to -12/-1. The list of probes is then pruned as follows. The first probe is kept. The second probe is compared with the first and kept only if its SW score is below a threshold of 160. The subsequent probes are successively compared with the list of already kept probes and added to this list if their SW scores are below 160, thus leaving a list of m probes.

#### 3. Scoring input sequences with probes

A comparison of all probes of the pruned collection *versus *all input sequences is performed using the SW algorithm with a Blosum62 scoring matrix and gap opening/extension penalties set to -12/-1. The SW scores *S *are normalised to obtain bitscores *S*_*bit *_[32] using:



The parameter values for the above used scoring system are λ = 0.312 and K = 0.076. These parameters were obtained by simulation with random sequences (see Materials and Methods). The bitscores are arranged in a matrix of dimensions Nxm.

#### 4. Protein distances

The above matrix is first transformed to a binary matrix based on a threshold of 27 (equivalent to a p-value of 0.01 [33,34]). All bitscores below this threshold are set to 0 and those above, to 1 (match). Since the binary variables are asymmetric, a distance matrix between any pair of input sequences represented by the rows of the binary matrix is calculated using the Jaccard distance [35]:



where n_11 _is the number of probes which match both sequences, and n_10 _and n_01 _are the number of probes, which only match one of the two sequences. n_00 _is excluded from the calculation of the distance (see below). This distance measure ranges from 0 (closely related sequences with all probes in common) to 1 (sequences with no probe in common).

#### 5. Identification of independent groups

One can identify groups that are separated by a distance of 1 (no match in common). These groups are called independent groups. The set of N input sequences is split into n independent groups containing l_i _proteins (1≤i≤n).

#### 6. Partitioning of proteins within an independent group

This is done using the PAM (Partitioning Around Medoids) algorithm [36] which, based on the above distance matrix, calculates all possible partitions ranging from 2 to l_i_-1 subgroups. For each partition the overall average silhouette width [37] is calculated and the partition that maximises it is considered optimal.

#### 7. Hierarchical clustering

The protein sequences are clustered using average-linkage agglomerative hierarchical clustering based on the Jaccard distances. The resulting tree is particularly useful to establish the relations between subgroups.

#### 8. Identification of diagnostic probes

For each probe the number of subgroups, to which it matches, is determined. As a result one can distinguish probes that only match one group, probes that match all groups (conserved regions found in all members of an independent group) and those in between.

### Case 1: Prokaryotic lyases

A set of protein sequences that can legitimately be arranged into a multiple sequence alignment is first considered to facilitate the comparison of the outcome of the JACOP protocol with other classification methods. All prokaryotic sequences retrieved with the Pfam (Version 7.3) HMM Lyase_1 (PF00206) in Swiss-Prot release 36 were retained as a test set. These are enzymes involved in double bond isomerisation, and catalyse five different reactions. A short sequence flanking a conserved methionine, described by the PROSITE pattern PS00163, is also present in all of these sequences.

**Figure 2:**
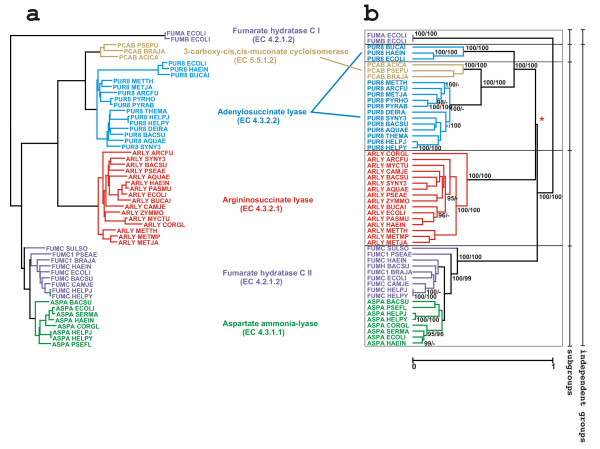
This figure shows the trees obtained either with ClustalW/PHYLIP (a) or with the JACOP protocol (b). The families of enzymes with different activities are presented in different colours. The resulting independent groups and subgroups found by JACOP are indicated by frames. In the case of the tree obtained with the JACOP protocol, the resampling/bootstrap values above 95% are indicated. The separation (*) between the three subgroups of homologues received a relatively low value because of comparable distances that induce a competition of sub-tree topology at that node.

Applying the JACOP protocol to this set of 53 sequences with an average length of 463 residues, resulted in the generation of 1473 probes to obtain three-time coverage. Only 154 probes were left after pruning, owing to the relatively high similarity among these sequences. The sequences were hierarchically clustered and the resulting tree is shown in Figure 2b. Two independent groups were obtained. The two class I fumarate hydratases are separated from all other sequences by a distance of one, i.e. the two groups do not share any probe and this despite the matches determined with the Pfam HMM and the PROSITE pattern. It turned out that these class I fumarate hydratases are biochemically different [38] (contain a [4Fe-4S] cluster, form homodimers and work under aerobic conditions) from the class II fumarate hydratases found among the remaining 51 sequences that form the second independent group (work under anaerobic conditions, do not contain an iron-sulfur cluster and form homotetramers). This latter group is partitioned into four sub-groups.

1. The argininosuccinate lyases form a dense cluster with a distinctive enzymatic activity.

2. The adenylosuccinate lyases (PUR8_*) with the exception of PUR8_ECOLI/BUCAI/HAEIN, which appear to be a separate sub-group, and the 3-carboxy-cis, cis-muconate cycloisomerase (PCAB_*) are clustered together.

3. The adenylosuccinate lyases PUR8_ECOLI/BUCAI/HAEIN.

4. The class II fumarate hydratases and aspartate ammonia-lyases are clustered together.

**Figure 3:**
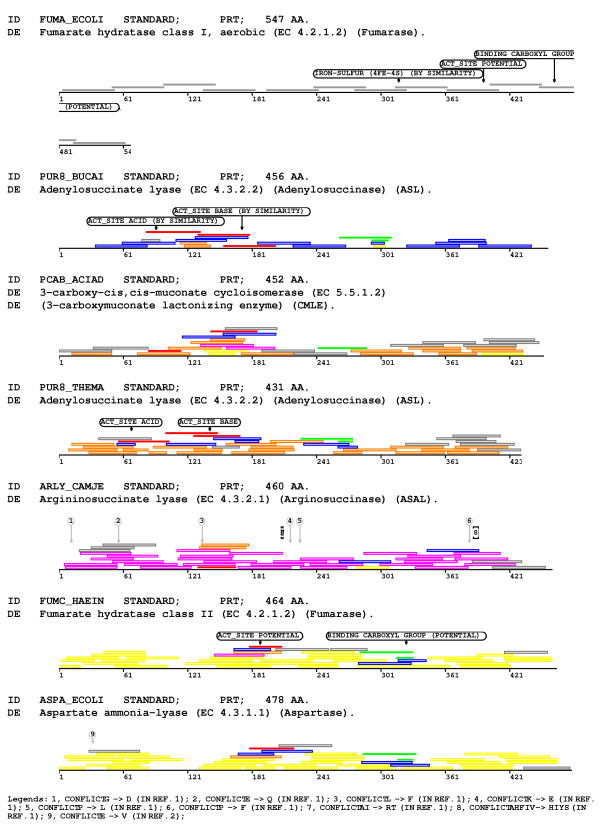
Representative proteins of the prokaryotic lyases are shown together with matching probes at their respective positions. The probes mapping to the representative of the first independent group (FUMA_ECOLI) are represented as closed dark grey bars. Probes that map to the active sites of proteins of the second independent group are shown as closed red bars, and those that map the region identified by the Pfam HMM Lyase_1 (PF00206) as closed green bars. Probes that map specifically to the different subgroups are depicted as open bars using the following colours: blue, orange, magenta and yellow. Probes that are not specific for any particular subgroup are depicted as grey open bars. Features were obtained from the original Swiss-Prot annotation.

Two sets of probes match to conserved regions of the second independent group (Figure 3). The first region corresponds to the active site according to Swiss-Prot annotation. The second region is the one identified with the Pfam HMM Lyase_1 (PF00206). In addition to these probes that identify conserved regions, other probes that are specific for the different subgroups were found.

The JACOP results were compared to the classification obtained using other approaches. Thus the sequences were aligned using ClustalW with default settings, and a tree was derived from this alignment using the PROTDIST and FITCH programs of the PHYLIP package [39] (Figure 2a). The trees are comparable, with differences implicating the problematic PUR8_ECOLI/BUCAI/HAEIN. However, the ClustalW/PHYLIP approach cannot provide any indication that the class I fumarate hydratases are unrelated to all other sequences. A bootstrap analysis on the multiple sequence alignment was also performed [40-42]. The results were rather confusing. Indeed a classical bootstrap strategy is designed to handle multiple sequence alignments of related sequences and is therefore unsuited to deal with such divergent sequences. As an alternative, the sequences were aligned using T-Coffee [43] with default parameters and the tree was established as before. In contrast to ClustalW, this tree confirmed the result found with JACOP (details not shown).

Furthermore, the classification obtained with JACOP was compared with other publicly available large-scale protein clusters. The COG (release 3) classification [25] was fully consistent with the enzymatic nomenclature and correctly separated the fumarate hydratases into two families. More surprisingly, it distinguished the 3-carboxy-cis, cis-muconate cycloisomerases from all the adenylosuccinate lyases. Unfortunately, details leading to this distinction were not available at the time of writing.

In the case of SYSTERS (release 4) [24], ProtoNet (version 4.0) [27] as well as ClusTr [28], the same 5 clusters were found.

### Parameter selection for JACOP

The rationale for our choices was the following:

i. Rigorous and well-described methods (SW algorithm, PAM algorithm) were preferred over faster but intricate heuristics for the sake of reproducibility by others. Nevertheless we tested BLAST (version 2.2.5) to compute the scores. However, this heuristic often failed to provide the correct SW scores, possibly due to the short length of the probes (details not shown).

ii. The main reason for choosing the PAM algorithm for partitioning the proteins was that this method, which is based on the minimization of the sum of dissimilarities, is more robust than methods that minimize the error sum of squares like k-means [36]. As an alternative to the PAM algorithm we have tested the fast TRIBE-MCL [22] algorithm but it failed to identify the relevant groups.

iii. Silhouette widths allow a good characterisation of all clusters that are not too elongated and make it possible to identify outliers in most situations. Another advantage of silhouette widths is their independence of the used partitioning algorithm. Silhouette widths s(*i*) [37] are calculated for each object *i *and range from -1 to +1. Values of s(*i*) close to one, indicate that the average dissimilarity of *i *to the other objects of the same cluster is much smaller than the smallest average dissimilarity to other clusters. If the value s(*i*) is about zero, then the two dissimilarities are approximately equal and hence it is not clear to which cluster the object *i *should be assigned. The worst situation takes place, when s(*i*) is negative indicating that object *i *has been misclassified. Furthermore, the overall average silhouette width over all objects can be used to objectively identify the most consistent partitioning for which it is the largest. The result of the partitioning is a list of protein sequences with the number of the subgroup to which they belong. Additionally, for each protein sequence, the number of the closest alternative subgroup and the corresponding silhouette width s(*i*) is given.

iv. Due to the random generation of the probes, one has to sample a sufficient number of them to "cover" the complete sequence. Sampling was stopped once the cumulated length of the probes exceeded three times the cumulated length of the input sequences (coverage 3x) because, in average, higher numbers do not further change the final number of probes after pruning.

v. The main reason for choosing the Jaccard distance measure was that it did not take into account non-significant matches shared by two proteins. As a consequence, proteins with no similarity other than noise are not grouped together.

The choice of the following parameters was based on two different validation procedures. The first one consists of re-sampling, i.e. the whole protocol is repeated 100 times from probe sampling to the partitioning of the independent groups, each time with a different seed for the random number generator. The second test is a classical bootstrap on the pruned probes, i.e. the first 3 steps of the protocol are run once and the resulting bitscore matrix is bootstrapped 100 times followed by the partitioning of the independent groups. The reproducibility of the 100 obtained partitions was evaluated based on the adjusted rand index [44]. It is a statistic designed to assess the degree of agreement between two partitions. An adjusted Rand index of 1 indicates identical partitions, whereas an adjusted rand index close to zero indicates random partitioning. After the simulations, each of the 100 partitions was compared to the other 99 partitions and the average was taken. The average adjusted rand indexes are given in Table 1 for the different parameter sets tested.

**Table 1:** This table summarizes the outcome of 100 resampling or bootstrapping tests done using the two data sets (prokaryotic lyases and SH2 containing proteins) with different parameter combinations. For each simulation the average adjusted rand index [44] has been calculated. The row marked in italic bold corresponds to the parameters used in the reference protocol. The values marked in bold indicate the changes made compared to the reference protocol.

				**Prokaryotic lyases**		**SH2 containing proteins**	
**Probe length**	**Pruning threshold**	**Coverage**	**Scoring system**	**Resampling**	**Bootstrap**	**Resampling**	**Bootstrap**

							
**25**	160	3	Blosum62/-12/-1	0.959	0.991	0.844	0.854
***50***	***160***	***3***	***Blosum62/-12/-1***	***1***	***1***	***0.932***	***0.937***
**100**	160	3	Blosum62/-12/-1	0.969	0.887	0.689	0.735
							
50	**40**	3	Blosum62/-12/-1	0.971	0.810	0.777	0.732
50	**80**	3	Blosum62/-12/-1	1	0.982	0.753	0.803
50	**120**	3	Blosum62/-12/-1	1	1	0.895	0.897
***50***	***160***	***3***	***Blosum62/-12/-1***	***1***	***1***	***0.932***	***0.937***
50	***200***	3	Blosum62/-12/-1	1	1	0.948	0.935
							
50	160	**1**	Blosum62/-12/-1	1	0.991	0.919	0.812
50	160	**2**	Blosum62/-12/-1	1	0.996	0.878	0.937
***50***	***160***	***3***	***Blosum62/-12/-1***	***1***	***1***	***0.932***	***0.937***
							
50	160	3	**Blosum45/-13/-3**	1	0.985	0.939	0.839
***50***	***160***	***3***	***Blosum62/-12/-1***	***1***	***1***	***0.932***	***0.937***
50	160	3	**Blosum80/-10/-1**	0.999	0.991	0.909	0.859

vi. Sampling with three times coverage results in a lot of redundancy. Eliminating probes that are too similar significantly reduces this redundancy, while keeping the information. Several threshold scores (raw scores of 40, 80, 120, 160 and 200) for the elimination of similar probes were tested for the pruning step. At a threshold of 40, most probes were eliminated and consequently most of the information. The resulting set of probes did not allow the robust and reproducible identification of subgroups. On the other hand, starting from threshold 160, the partitions were reproducible. In all cases the gain in resolution and information was marginal when the threshold was increased to 200. In addition, pruning has the desirable side effect that it preferentially removes probes with low complexity regions [45,46] – which are known to be a nuisance in sequence comparison.

vii. Because of the use of a local alignment algorithm, the average length of a match was shorter than the length of the probes (average length of about 19 for the above set of sequences). This implies that relatively long probes could document short conserved motifs. The use of probes of length 25 or 100 resulted in a substantially decreased reproducibility.

viii. The similarity matrices and gap penalties for the SW algorithm parameters were selected to ensure that the scoring system produces true local alignments (logarithmic phase [47]; this allowed the use of well established statistics [47-53].In addition to the scoring system Blosum62/-12/-1, the Blosum45/-13/-3 and the Blosum80/-10/-1 scoring systems were also evaluated with the appropriate values for λ and K. Surprisingly this only had a marginal effect on the outcome.

ix. Hierarchical clustering was also applied to the data sets generated during the validation procedure. The resulting 100 trees were compared to determine the stability of the nodes. In the case of prokaryotic lyases, counts larger than 95 are reported on the tree of Figure 2b. The outcomes of the two validation procedures agree to a large extent. Interestingly, the separation between the three major sub-groups of homologues received a relatively low value because comparable distances separated them. This results in a competition of sub-tree topology at that node. This incidentally indicates the superiority of the PAM algorithm over hierarchical clustering for our purpose.

### Case 2: SH2 containing proteins

Here a set of protein sequences that cannot be arranged as a meaningful multiple sequence alignment is considered. This set contains all proteins from Swiss-Prot (release 40) with at least one Src homology 2 (SH2) domain as predicted by the Pfam HMM PF00017. The 203 proteins of this set belong to the super-family of intracellular signal-transducing proteins and represent a case study of modular architecture [17]. Indeed, together with one or two SH2 domains, many other domains were found. Amongst them are RhoGAP, RhoGEF, protein-tyrosine kinases (PTK), protein-tyrosine phosphatase or phosphatidylinositol specific phospholipase C X or Y domains, as well as the promiscuous SH3 domain [22]. In addition, 5% of all residues were found to be part of low complexity regions by the SEG program [45].

The JACOP reference protocol was applied to this set of 203 sequences of an average length of 628 residues. 7647 probes were extracted to obtain three-time coverage and 1799 probes remained after pruning. JACOP identified one single independent group containing 75 subgroups, which correlate very well with the Swiss-Prot IDs. Also the domain architectures – as identified by the Pfam HMM – correlate well with the subgroups identified in that every subgroup is reflected by one single domain architecture. However, different subgroups may share the same architecture. At this stage the result of the hierarchical clustering becomes helpful to delineate relationships among subgroups. That way one can distinguish three larger superfamilies (together with two singletons, three pairs and one quadruplet) (not shown).

**Figure 4:**
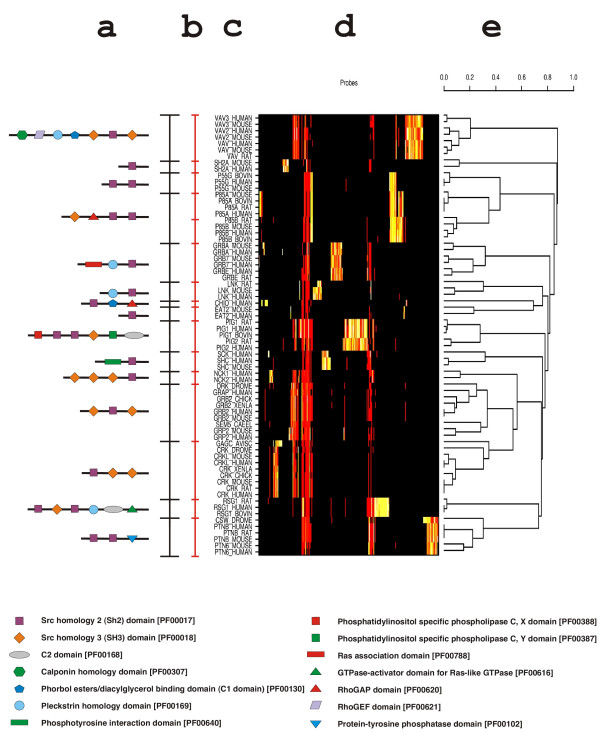
Classification of 69 SH2-containing protein sequences that represent various domain architectures. a: domain architecture as predicted by the Pfam HMMs; b: partitioning obtained by JACOP; c: Swiss-Prot IDs; d: the bitscore matrix with a colour code based on heat colours ranging from white for high values to red for low values, plus black for all bitscores that are below 27; e: the tree obtained by hierarchical clustering of the proteins based on Jaccard distances.

• 24 signal transduction and activators of transcription (STAT) proteins are present in the first superfamily. They are subdivided into 7 subgroups. However, all 24 proteins share the same function and domain architecture together with a single SH2 domain (details not shown).

• 98 proteins containing a protein-tyrosine kinase (PTK) motif (PF00069) make up the second superfamily and are subdivided into 36 subgroups. The number of different architectures was limited, the two most frequent being SH3-SH2-PTK and SH2-PTK-PTK (details not shown).

• The third superfamily, containing 69 proteins, is the most complex one as the domain architectures found are extremely diverse. 15 subgroups are identified and represent 14 different architectures (Figure 4). The domain architectures – as identified by the Pfam HMM – correlate well with the subgroups identified. This superfamily is functionally diverse and contains enzymes, adaptor proteins, docking proteins and regulatory proteins [17]. Two sets of proteins deserve further discussion:

1. The phosphatidylinositol 3-kinase regulatory alpha (P85A_*), beta P85B_*) or gamma (P55G_*) subunits share two SH2 domains at the C-terminus [54]. In addition, the P85 subunits contain an SH3 and a RhoGAP domain at the N-terminus. Despite the different architecture, these sequences were clustered together due to the presence of highly conserved tandem SH2 domains (sequence identity greater than 70%), which were found to be quite distinct from tandem SH2 domains found in other subgroups.

2. The SH2/SH3 containing adaptors (NCK*_*, DRK_DROME, GRAP_HUMAN, GRB2_*, SEM5_CAEEL, GRP2_*, GAGC_AVIS and CRK*_*) are subdivided into 4 subgroups corresponding to different arrangements of the SH2 and SH3 domains. Their SH2 or SH3 domain sequences are more similar to each other than to the SH2/SH3 domain sequences found in other subgroups (details not shown). This strongly suggests, that these proteins were subject to a recent reshuffling event. Interestingly, the common denominator of these proteins is their role in regulating tyrosine kinase signalling. They serve to recruit proline-rich effector molecules to tyrosine-phosphorylated kinases or their substrates [55] and references therein).

As for the prokaryotic lyases, the probes were analysed. The grouping of the probes can be seen in Figure 4d. Several groups of probes that are specific for particular subgroups can easily be distinguished from probes that are of more general nature and map to regions that are conserved amongst all proteins of this superfamily. However, probes that are specific for one subgroup and that appear as a block in Figure 4d do not necessarily map to adjacent regions but can be distant from one another. The only common denominator of such probes is the fact that they match the same proteins.

The results for the third superfamily were compared to SYSTERS, ProtoMap, ProtoNet as well as ClusTr. The classifications of those approaches were found to be very similar to the partitioning obtained by JACOP.

## Conclusion

The key point of the JACOP protocol is the random sampling of relatively short sub-sequences (probes) out of the sequences to be analysed. After a normalisation step, the probes are compared with the initial set of proteins and the resulting scores are used to classify the proteins based on a p-value of 0.01. The method produces meaningful and robust partitions of proteins with related functions out of a set of input sequences, even when the sequences cannot be arranged in a multiple sequence alignment due to their modular architecture and despite the method's stochastic nature. It also allows the identification of regions conserved amongst all sequences of an independent group or, alternatively, regions that are specific (diagnostic) to certain subgroups.

In our opinion one of the reasons for the robustness of JACOP is the use of the complete information present in the pool of pruned probes. In contrast to other methods that are based on direct pairwise comparisons, JACOP also uses information on protein sequence similarities outside the protein's own group. Hence a distance between two proteins is based on how similar the two proteins are with respect to some features present in the pool represented by the pruned probes and how dissimilar they are to other features.

It would seem appealing to define probes using the natural boundaries of the protein domains, in an attempt to describe the protein sequences by a systematic tiling with probes, in the same spirit as ProDom [9]. Unfortunately, there is no reliable algorithm for detecting domain boundaries. Also, whether they can be defined unambiguously is still an open question. However, it is obvious that a false definition of the boundaries of a domain has direct consequences on the definition of the boundaries of the adjacent domains if probes do not overlap. In this perspective, random sampling of potentially overlapping probes – in contrast to a systematic tiling of probes – appears to be a simple way to produce a set of sub-sequences with unbiased positions and boundaries. In addition, bootstrapping and/or re-sampling may be performed to estimate the stabilities of the resulting partitions.

Residue substitutions are certainly, and by far, more frequent in the course of evolution than insertions or deletions, which themselves are far more frequent than domain architecture re-organization. However, these different types of events co-occur and some domain re-organizations may be expected to have occurred more recently than many residue substitutions. This usually causes major problems in most methods that use direct pairwise alignments of two sequences to measure their similarity. The introduction of a collection of unordered probes as an intermediary layer in the comparison of two sequences elegantly solves the problem. Hence, when comparing a pair of sequences that exemplify a case of domain swapping, the sequences are locally co-linear through the probes, everywhere but in the swap region itself. This strategy results in a partial uncoupling of the domain architecture present in the proteins.

A Jaccard distance of 0.5 between two sequences (eq. 2) can actually correspond to different cases. The sequences can be globally homologous but sufficiently divergent to share only one half of the probes. Alternatively, one of the sequences can be a perfectly conserved fragment of about half the length of the other sequence. Hence, the JACOP method includes an implicit weighing scheme that relates the similarity measure at the sequence level to the architecture similarity. A better understanding and control of the implicit weighing scheme is the subject of future work.

When comparing the results of JACOP with other publicly available automated classifications, our results closely resembled the ones proposed by reference classifications such as Systers and ProtoMap. However – though simple – JACOP is a robust, efficient and reproducible approach for the classification of protein sequences. Also, JACOP can easily be applied since it only requires software (Perl [56], ssearch [57] and R) and algorithms (SW, PAM) available to every one.

## Materials and methods

### Implementation

The first 3 steps of the JACOP protocol, presented below, were performed using scripts written in Perl 5 [56]. Calculation of the raw Smith-Waterman (SW) scores [58] was either done using the program ssearch [57] or hardware accelerated using a GENEMATCHER (Paracel, Pasadena, CA). All statistical calculations were done using the statistics software R [59]. A package for R was specifically written for JACOP and the source code is available upon request.

### Statistical parameters

Statistics for the scores of local alignments, unlike those of global alignments, are well understood [33,34,47-52,60-62]. The parameters for the underlying extreme value distribution (EVD) for the three scoring systems (Blosum62/-12/-1, Blosum45/-13/-3 and Blosum80/-10/-1) were estimated using random protein sequences of appropriate lengths. The SW scores obtained were subsequently used to estimate the EVD parameters by maximum likelihood [63].

## Availability and requirements

Project name: JACOP; Project home page:  Operating system(s):Platform independent.

## Authors' contributions

PS developed and implemented the JACOP approach. MP conceived the method and provided guidance.
